# A Retrospective Multicenter Analysis of the Incidence of Bone-Only Disease at PSMA PET/CT in Castration Resistant Prostate Cancer Patients

**DOI:** 10.3390/cancers15082208

**Published:** 2023-04-08

**Authors:** Francesca Serani, Wolfgang P. Fendler, Paolo Castellucci, Christoph Berliner, Francesco Barbato, Ken Herrmann, Andrea Farolfi, Stefano Fanti

**Affiliations:** 1 Department of Medical and Surgical Sciences (DIMEC), Alma Mater Studiorum University of Bologna, 40126 Bologna, Italy; stefano.fanti@aosp.bo.it; 2 Department of Nuclear Medicine, University Hospital Essen, University of Duisburg-Essen, 45147 Essen, Germany; 3 Nuclear Medicine Department, IRCCS Azienda Ospedaliero—Universitaria di Bologna, 40138 Bologna, Italy

**Keywords:** prostate cancer, CRPC, bone metastasis, metastasis-directed therapy

## Abstract

**Simple Summary:**

This study aimed to determine how often castration-resistant prostate cancer spreads only to the bones and to identify factors that predict this spread. The researchers looked at PSMA PET scans from 179 men. They found that 20% had cancer that had spread only to their bones, with the most common sites being the spine, ribs, and hips. Men who were diagnosed with lymph-node spread at diagnosis or who had only received hormone treatment were more likely to have bone-only spread. These findings suggest that men with bone-only disease could benefit from personalized treatment that specifically targets the bone. The study highlights the importance of PSMA PET scans in accurately detecting prostate cancer and helping doctors choose the best treatment.

**Abstract:**

PSMA PET/CT has unprecedented accuracy for localization of initial or recurrent prostate cancer (PC), which can be applied in a metastasis-directed therapy approach. PSMA PET/CT (PET) also has a role in the selection of patients for metastasis-directed therapy or radioligand therapy and therapy assessment in CRPC patients. The purpose of this multicenter retrospective study was to determine the incidence of bone-only metastasis in CRPC patients who underwent PSMA PET/CT for restaging, as well as identifying potential predictors of bone-only PET positivity. The study analyzed data from 179 patients from two centers in Essen and Bologna. Results showed that 20.1% of the patients had PSMA uptake only in the bone, with the most frequent lesions located in the vertebrae, ribs, and hip bone. Half half of the patients showed oligo disease in bone and may benefit from a bone-metastasis-directed therapy. Initial positive nodal status and solitary ADT were shown to be negative predictors of osseous metastasis. The role of PSMA PET/TC in this patient population needs to be further explored in terms of its role in the evaluation and adoption of bone-specific therapies.

## 1. Introduction

Prostate cancer (PC) is a major cause of morbidity and mortality in men worldwide; in the United States, approximately 34,500 prostate cancer deaths have been estimated for 2022, a significant proportion of which were at the metastatic stage [1]; specifically, bone disease is difficult to treat and comes with considerable reduction in quality of life and survival [2]. In Europe, although the trend for prostate cancer specific mortality is decreasing [3], it is still a high burden disease.

PSMA PET/CT, both with 68Gallium or 18Fluorine, has shown unprecedented accuracy for localization of initial or recurrent prostate cancer [4], and it is now a widely used imaging technique in the clinical setting given its high accuracy for staging [5,6] and restaging of patients with PC in a biochemical relapse (BCR) context [7,8,9].

The introduction of PET/CT with more specific tracers for prostate cancer (Choline, Fluciclovine and PSMA) led to a metastasis-directed therapy (MDT) approach, to delay systemic therapy [8,10,11]. There are still limited data available regarding the outcomes consequent on MDT guided by PSMA PET/CT imaging [12], but there is hope this imaging technique can lead to an improvement of overall survival (OS) and progression-free survival (PFS) in these set of patients.

PSMA PET/CT is increasingly being utilized for both initial staging and restaging of prostate cancer, including in patients with castration-resistant prostate cancer (CRPC) [13], and for identifying candidates for radioligand therapy with Lu-PSMA. However, the clinical significance of PSMA PET/CT findings in patients with CRPC remains uncertain. As stated in the EAU guidelines, all the therapeutic recommendations are based on conventional imaging only [14]. Patients with metastatic CRPC (mCRPC) and osseous predominance can benefit from bone-specific therapies both as active treatment and as protective agent for symptomatic skeletal events: The only approved bone-specific drug that is associated with a survival benefit is [223Ra]RaCl2 (Ra223). In 2018, the European Medicines Agency (EMA) concluded its review of Ra223, and given safety issues, has recommended restricting its use to patients who have had two previous treatments for metastatic prostate cancer [15]. The role of PSMA PET/CT in this setting is still unclear.

To avoid skeletal-related events in patients with bone metastasis, various treatments can be used, such as radiotherapy on the bone lesion and the use of bisphosphonates and RANK ligand inhibitors [14]. For RANK ligand inhibitors, in particular, in non-metastatic CRPC (nmCRPC) for conventional imaging, denosumab has been associated with increased bone-metastasis-free survival compared to placebo [16].

Given the high predominance of bone metastasis in CRPC patients and their possible exclusive presence [17], with the current study we wanted to retrospectively analyze the incidence of bone-only PSMA uptake in a population of consecutive CRPC patients who underwent PSMA PET/CT for restaging. The secondary endpoint of the study was to evaluate the presence of potential predictors of bone-only PSMA PET/CT positivity.

## 2. Materials and Methods

This is a multicentric, retrospective observational cohort study in patients with CRPC—defined in accordance with the EAU guidelines [14]: castrate serum testosterone < 50 ng/dL plus either biochemical progression, defined as PSA increase, or radiological progression, defined as the appearance of new lesions either on bone scan or soft tissue lesion at CT—which consecutively performed their first 68Ga-PSMA PET/CT between January 2019 and December 2019 in two high-volume Nuclear Medicine centers (Essen, Germany, and Bologna, Italy). A total of 68Ga-PSMA PET/CT images were reviewed according to PROMISE Criteria [18].

A univariate analysis with Fisher analysis was performed between bone-only metastasis population and other CRPC patients for age (at diagnosis and at PSMA PET/CT), nodal involvement at primary treatment, ISUP, iPSA, PSA, PSAdt and PSAvel, time elapsed between primary treatment and the PET scan and number of systemic treatments performed during CRPC status before PET.

All patients gave written informed consent to undergo PSMA PET. This retrospective study was approved by the respective ethic review boards (Essen: 19-8570-BO; Bologna: 244/2016/O/Oss) and necessity for study specific consent was waived.

## 3. Results

A total of 179 patients were considered for analysis (65 from Bologna and 114 from Essen); 36 out of 179 patients (20.1%) showed PSMA uptake only in the bone.

Of these 36 patients, the median age at diagnosis was 64 y (IQR, 58.0–69.0 y), median age at PET/CT was 74.0 y (IQR, 67.0–78.3 y), median ISUP grade was 4 and median iPSA was 10.0 ng/mL (IQR, 6.3–36.9 ng/mL). Median PSA before PET/CT was 5.16 ng/mL (IQR, 1.5–12.0 ng/mL), PSAdt 2.8 months (IQR 2.0–5.1 months) and PSAvel 8.4 ng/mL/year (IQR 2.9–35.9). Median time elapsed between primary treatment and the PET scan was 6 years (IQR 4–10) and the median number of systemic treatments performed during CRPC status was 1 (Summarized in Table 1).

In a per-patient analysis, 50.0% of the patients (18/36) showed focal PSMA uptake in three lesions or fewer (oligometastatic), 41.7% (15/36) showed focal PSMA uptake in more than three lesions, and 8.3% (3/36) showed a diffuse bone marrow PSMA uptake.

In Table 2, it is interesting to note that out of the 18 patients with oligometastatic disease, 6/18 (33.3%) had a focal uptake in only one rib with either a PSMA uptake greater than the liver or a corresponding CT correlate. It can also be noted that 72.2% of the PSMA uptake of most representative lesions corresponded to CT pathological sclerotic findings while 8.3% had no corresponding finding on CT, and 19.4% were in correspondence of lytic lesions on CT.

In a per-patient analysis, the areas of increased PSMA uptake were localized: 30.6% (11/36) sacrum (median SUVmax 19.2 g/mL, IQR 8.9–24.3); 44.4% (16/36) hip bone (median SUVmax = 23.6 g/mL, IQR 12.5–24.3); 55.6% (20/36) vertebrae (median SUVmax = 23.5 g/mL, IQR 16.4–35.8); 36.1% (13/36) sternum (median SUVmax = 13.2 g/mL, IQR 6.7–23.9); 55.6% (20/36) ribs (median SUVmax = 13.8 g/mL, IQR 7.8–79.3); 22.2% (8/36) femur (median SUVmax = 16.8 g/mL, IQR 9.0–23.7); 19.4% (7/36) humerus (median SUVmax = 14.9 g/mL, IQR 9.8–21.9); 27.8% (10/36) scapula (median SUVmax = 17.9 g/mL, IQR 9.9–24.6); 13.9% (5/36) clavicula (median SUVmax = 17.2 g/mL, IQR 8.1–23.3); and 13.9% (5/36) in the skull (median SUVmax = 15.2 g/mL, IQR 11.6–21.3) (summarized in Table 3).

Regarding the secondary aim of the study (predictors of bone-only PSMA PET/CT positivity), at the univariate analysis, we observed that pN1 patients at presentation were more likely to show bone-only disease at PSMA PET/CT (*p* < 0.01) if compared with other CRPC patients. Moreover, patients who received only ADT as primary treatment were more likely to show bone-only uptake at PSMA PET/CT when classified as CRPC (*p* < 0.01).

The other analyzed factors (age both at diagnosis and at PSMA PET/CT, ISUP, iPSA, PSA, PSAdt, and PSAvel, time elapsed between primary treatment and the PET scan and number of systemic treatments performed during CRPC status before PET) did not show any statistical significance. 

The *p*-values retrieved by the Fisher exact test are summarized in Table 4.

## 4. Discussion

To our knowledge, this is the first study evaluating the incidence of bone-only PSMA focal uptake in the CRPC population. In this analysis, the incidence of bone-only uptake was 20% of all the patients who received a PSMA PET during their CRPC status.

In a per-patient analysis, we found that half of the patients showed an oligometastatic spread of the disease only (three or fewer focal PSMA lesions, according to PROMISE Criteria [18]).

Two recent phase II randomized trials, ORIOLE [19] and STOMP [20], showed interesting results for MDT.

The ORIOLE used only stereotactic ablative radiation (SABR) in the treatment arm, whereas the STOMP trial included patients whose metastases were treated with different techniques, either radiotherapy or surgery.

Regarding this difference in treatments, both trials showed that for patients with oligometastatic hormone-sensitive PC, MTD is safe and improves androgen-deprivation-therapy (ADT)-free survival when compared to surveillance only.

However, neither of the studies used PSMA PET/CT as an inclusion criterion: STOMP used Choline PET/CT, whereas ORIOLE used conventional imaging. The latter one, for patients randomized in the treatment SABR arm, performed a PSMA PET/CT and the investigative team was blinded to the PSMA PET/CT results: this resulted in 16 out of 36 patients with PSMA avid lesions not being included in the treatment field [19]. The median progression-free survival (PFS) (rise of PSA or radiologic progression or initiation of ADT) was unreached among participants with no untreated lesions vs. 11.8 months among participants with any untreated lesions [19].

It has now been reported in the literature that PSMA PET/CT has a higher sensitivity than CT and bone scan, leading to a change in patient management [21,22], especially in the staging setting.

A recent retrospective multicenter study by Zamboglou et al. [23] used PSMA PET/CT to guide salvage radiotherapy, and the metastasis-free survival at 4 years was 83% of the 815 patients.

These findings support the use of PSMA PET/CT for MTD; the concept that oligometastatic disease may have a different biological potential without a fully developed metastatic power has arisen since a 1995 study by Hellmann et al. [24], and at this stage there may still be the possibility to have a curative intent for PC [19].

In this context, it may be hypothesized that these patients could benefit from an MDT approach, not only for pain control but in order to delay as much as clinically possible the introduction of advanced systemic therapies and their adverse reaction.

The studies cited, however, had a different population: they had a hormone-sensitive prostate cancer population, whereas our study investigated a CRPC population. To this day, it has not been investigated whether the CRPC population could benefit from an MDT approach.

In a per-patient analysis, roughly 40% of the patients showed a disseminated disease with more than three focal PSMA uptakes in bone segments; at this point of the disease, both for STOMP and for ORIOLE inclusion criteria [19,20], these patients should be considered for a systemic approach including the introduction of bone-protective agents such as bisphosphonates or RANK ligand inhibitors.

A total of 72.2% of patients had a PSMA uptake of the most representative lesion on sclerotic findings at corresponding CT images for patients with multiple symptomatic sclerotic bone metastases, therapy with 223Ra can be considered, according to the selection criteria of the phase III ALSYMPCA trial [25]. Patients eligible for 223Ra therapy have to show more than six lesions at bone scintigraphy, which is now well known to be less sensitive than PSMA PET/CT. In the ALSYMPCA trial, having fewer than six lesions on bone scintigraphy was an unfavorable risk for 223Ra therapy; it is not yet known if the use of PSMA PET/CT could better select the patients, given that having six bone lesions at PSMA PET/CT may not correspond to six lesions at bone scan. At the moment, there is only one registered clinical trial (NCT04951817) evaluating the change of lesion detection number at PSMA PET/CT in patients selected with bone scan for 223Ra therapy [26].

With the 2018 EMA safety concerns, the prescription for this therapy has to be crafted carefully, and future analysis of these concerns is needed.

A total of 8.3% of the patients had no corresponding pathological CT images underneath their most representative lesion, and the question is still open of what the best treatment option for patients without any morphological corresponding finding could be.

It is well known that PSMA PET/CT can have false-positive bone findings, which may lead to inadequate therapy; however, experienced nuclear medicine physicians are able to correctly detect and identify the lesions as unspecific [27].

It has already been shown that using PSMA PET/CT leads to an upstaging in a majority of CRPC patients non-metastatic by conventional imaging [28]. 

Fendler et al. [29] conducted a retrospective analysis of 200 PSMA PET/CT scans in patients with non-metastatic castration-resistant prostate cancer (nmCRPC), which revealed that 98% of the patients analyzed (196/200) had a positive result on PSMA PET/CT. Among these patients, 16% (31/200) were found to have metastatic disease without evidence of local or pelvic recurrence.

All these patients may benefit from a combined approach of MTD and bisphosphonates or RANK ligand inhibitors.

To this day, it is not clear if the use of bisphosphonates provided early in the course of cancer will be able to prevent the formation of bone metastases or otherwise [30].

A high PSMA uptake demonstrated by bone lesions shows that patients with disseminated bone disease could benefit from a bone-directed and/or PSMA-directed therapy, by VISION Criteria [13]. Patients with a high osseous metastatic burden in PSMA PET/CT have an overall worse prognosis and a higher risk of myelotoxicity when treated with 177Lutetium-PSMA-617 (Lu PSMA) [31]. However, the recent publication of an interim analysis of the RALU study shows the safety and feasibility of the sequential use of first Ra223 (Ra223 and then Lu PSMA [32], both myelotoxic therapies). Moreover, the findings from the WARMTH study showed that a prior therapy with 223Ra had a positive impact on overall survival in patients who were subsequently treated with LuPSMA [33]. Therefore, this patient population can potentially benefit from accurate selection by PSMA PET/CT.

Patients with a more advanced stage at diagnosis (pN1) or treated with ADT only were more likely to show bone-only PSMA uptake when a PSMA PET/CT was performed during their CRPC status. These patients may be considered at a higher risk of showing bone metastasis and may be considered for a more aggressive therapy since diagnosis. A systematic review investigating the role of neoadjuvant ARSI therapy in unfavorable intermediate- and high-risk prostate cancer showed that a pathological complete response is rarely attained but promising results are obtained in terms of positive surgical margins [34]. A more recent publication showed similar results [35] and is awaiting publications of clinical trials [36]. None of the studies cited had PSMA PET/CT as staging diagnostic criteria.

There are various limitations to our study. First, its retrospective design involved collecting clinical information from the nuclear medicine file’s notes and relying on the information provided by the clinician during the examination’s booking. Other limitations are the small sample size considered for analysis, heterogeneous CRPC population (both early and advanced), and lack of follow-up.

## 5. Conclusions

In this retrospective study, the incidence of PSMA PET/CT bone-only disease in a CRPC population was 20%, showing that PSMA PET/CT reliably identifies bone metastases in CRPC patients, opening the door for bone-targeted therapies and potentially improving treatment sequences. Initial positive nodal status and solitary ADT were shown to be negative predictors of osseous metastasis. The role of PSMA PET/CT in this patient population needs to be further explored in terms of its role in the evaluation and adoption of bone-specific therapies.

## Figures and Tables

**Table 1:** Patient characteristics.

Characteristic	Median	IQR
Age at diagnosis (y)	64.0	58.0–69.0
Age at PSMA PET/CT (y)	74.0	67.0–78.3
ISUP Grade	4	-
Initial PSA (ng/mL)	10.0	6.3–36.9
PSA before PET/CT (ng/mL)	5.16	1.5–12.0
PSA doubling time (months)	2.8	2.0–5.1
PSA velocity (ng/mL/year)	8.4	2.9–35.9
Time elapsed primary therapy to PSMA PET/CT (y)	6	4–10
Systemic treatment performed	1	-

**Table 2:** Per patient localizations of PSMA bone uptake.

Patient	Unifocal vs. Oligometastatic vs. Disseminated vs. Diffuse Bone Marrow Involvement	Number of Lesions	Location	Highest Suvmax	Ct Findings for Representative Lesions
1	uni	1	rib	9.3	lytic
2	uni	1	rib	4.5	sclerotic
3	diss	4	sacrum, sternum, ribs	11.9	sclerotic
4	oligo	2	vertebrae, rib	27	none
5	oligo	2	vertebrae	19.9	none
6	uni	1	skull	17.2	lytic
7	uni	1	rib	10	sclerotic
8	uni	1	rib	26.3	sclerotic
9	uni	1	vertebrae	25.4	sclerotic
10	diss	5	hip bone, femur, ribs	8.2	sclerotic
11	diss	7	hip bone, femur, homerus, scapula	8.2	sclerotic
12	oligo	3	sacrum, femur	10.3	sclerotic
13	diss	>10	hip bone, vertebrae, sternum	43.9	sclerotic
14	diss	>10	vertebrae, sternum, ribs, scapula	74.8	none
15	diss	10	hip bone, vertebrae, sternum, ribs, skull	28	sclerotic
16	diss	>10	sacrum, hip bone, femur, vertebrae, sternum, ribs, scapula, skull	70.2	sclerotic
17	uni	1	vertebrae	14.6	sclerotic
18	diss	>10	sacrum, hip bone, verterbrae, ribs scapula	29.9	lytic
19	diss	5	vertebrae, sternum,	30.2	sclerotic
20	oligo	2	sacrum, hip bone	13.2	sclerotic
21	dmi	diffuse bone marrow	diffuse	75.4	sclerotic
22	uni	1	hip bone	32.4	sclerotic
23	diss	>10	sacrum, hip bone, femur, vertebrae, sternum, ribs, scapula	15.4	sclerotic
24	diss	5	hip bone, femur, vertebrae	2.9	sclerotic
25	uni	1	hip bone	30.4	sclerotic
26	uni	1	rib	8.6	sclerotic
27	uni	1	hip bone	3.9	lytic
28	diss	>10	hip bone, vertebrae, sternum, ribs, homerus, scapula, clavicula	37.9	sclerotic
29	dmi	diffuse bone marrow	diffuse	22.9	sclerotic
30	oligo	2	vertebrae, hip bone	144.8	sclerotic
31	diss	6	vertebrae, sternum, ribs, homerus, scapula, clavicula	4.5	none
32	diss	>10	sacrum, hip bone, femur, vertebrae, sternum, ribs, scapula	167.7	sclerotic
33	uni	1	sacrum	26.9	lytic
34	dmi	diffuse bone marrow	diffuse	25.9	sclerotic
35	uni	1	rib	3.9	sclerotic
36	diss	5	hip bone, vertebrae, ribs	43.9	lytic

Uni = unifocal uptake; Oligo = oligometastatic disease; Diss = disseminated disease; Dmi = diffuse bone marrow involvement.

**Table 3:** Summary of per-patient localization and SUVmax.

Location	% of Patients (*n*)	Median SUVmax (g/mL)	IQR (g/mL)
Vertebrae	55.6% (20/36)	23.5	16.4–35.8
Ribs	55.6% (20/36)	13.8	7.8–79.3
Hip bone	44.4% (16/36)	23.6	12.5–24.3
Sternum	36.1% (13/36)	13.2	6.7–23.9
Sacrum	30.6% (11/36)	19.2	8.9–24.3
Scapula	27.8% (10/36)	17.9	9.9–24.6
Femur	22.2% (8/36)	16.8	9.0–23.7
Humerus	19.4% (7/36)	14.9	9.8–21.9
Clavicula	13.9% (5/36)	17.2	8.1–23.3
Skull	13.9% (5/36)	15.2	11.6–21.3

**Table 4:** Univariate analysis of the variables taken into consideration.

Variable	Fisher’s Exact Test *p* Value
pN1 at primary treatment	0.009
ADT as single therapy	0.005
Age at diagnosis	0.050
Age at PSMA PET/CT	0.045
ISUP	0.535
initial PSA	0.242
PSA at PSMA PET/CT	0.326
PSAdt	0.829
PSAvel	0.526
Time to PSMA PET/CT from primary treatment	0.045
Number of systemic treatments performed	0.444

## Data Availability

Data are not available due to privacy and ethics restrictions.
